# A comprehensive evaluation of the antimicrobial efficacy of concentrated growth factor against biofilms associated with endodontic diseases: An in vitro study

**DOI:** 10.4317/jced.63049

**Published:** 2026-01-28

**Authors:** Negin Firouzi, Bahar Asheghi, Ramtin Chitsazha, Maryam Ayoobi, Mohammad Mehdi Karimi Mazidi, Maryam Pourhajibagher

**Affiliations:** 1 Department of Endodontics, School of Dentistry, Shiraz University of Medical Sciences, Shiraz, Iran; 2 Department of Periodontics, School of Dentistry, Shiraz University of Medical Sciences, Shiraz, Iran; 3 Student Research Committee, School of Dentistry, Shiraz University of Medical Sciences, Shiraz, Iran; 4 Dental Research Center, Dentistry Research Institute, Tehran University of Medical Sciences, Tehran, Iran

## Abstract

**Background:**

Concentrated Growth Factor (CGF), derived from a specific centrifugation protocol designed to optimize the concentration of growth factors and platelet-derived cytokines, has been investigated in the context of regenerative treatments. However, the potential antibiofilm properties of CGF against endodontic pathogens remain to be clarified. This in vitro study evaluated the antimicrobial effectiveness of CGF against the biofilms of Enterococcus faecalis and Candida albicans.

**Material and Methods:**

Blood samples were collected from otherwise healthy volunteers. A dedicated centrifugation device and protocol were used for CGF preparation. The antimicrobial activity of the CGF was observed and recorded against standard strains of E. faecalis and C. albicans using a disc agar diffusion method to determine the inhibition zone, and broth microdilution to measure the minimum inhibitory concentration (MIC) and minimum bactericidal concentration (MBC). A crystal violet assay and colony-forming unit (CFU) counts were conducted for biofilm assessment, using sodium hypochlorite (NaOCl) 5.25% as a positive control. This was followed by scanning electron microscopy (SEM) to analyze the morphology of the biofilm formed on the dentinal surface. A statistical analysis was performed using one-way ANOVA followed by Tukey's multiple comparison test with GraphPad Prism (version 8.4.3).

**Results:**

An inhibition zone was observed in both the CGF and NaOCl groups against both microorganisms. MIC was found for CGF against both microorganisms at a concentration of 50% v/v, while MBC and MFC were obtained at a concentration of 100% v/v. CFU counting revealed a significant reduction in viable microbial cells following the treatment of E. faecalis and C. albicans biofilms with NaOCl and CGF compared to the control group (P&lt;0.05). NaOCl resulted in the most pronounced reduction of the biofilms (76.86% for E. faecalis and 86.52% for C. albicans). However, there was a 32.97% reduction of the viable microbial cells of E. faecalis and a 35.97% reduction of the viable microbial cells of C. albicans in biofilm treated with CGF. The SEM results also showed a notable decrease in the concentration of fungal and bacterial cells.

**Conclusions:**

The present study demonstrated that CGF may exhibit antimicrobial and antibiofilm properties against endodontic pathogens. Hence, CGF may contribute to reducing microbial load and enhancing the outcome of regenerative treatments within infected root canal systems.

## Introduction

Pulp necrosis in immature teeth disturbs root development, which, due to the presence of thin dentinal walls, increases the risk of the cervical fracture of the root, particularly when less than half of the root is developed (1 , 2). In such cases, regenerative treatments help the biological replacement of tooth structures, including dentin, root, and pulp-dentin complex cells. Regenerative endodontic treatment (RET) relies on three fundamental components: tissue-forming stem cells, a conductive environment, and signaling molecules (3). Two main strategies have been considered for pulp regeneration: cell homing and cell transplantation. In the cell transplantation method, exogenous stem cells are seeded into scaffolds enriched with signaling molecules (4). In contrast, the cell-homing approach relies on recruiting endogenous stem cells, guided by biological signaling molecules, to the target site (5). Traditionally, the cell-homing method has involved intentionally inducing bleeding from periradicular tissues by over-instrumentation. The resulting blood clot serves as a natural scaffold for the incoming stem cells (6). However, this method has several limitations, including challenges in achieving adequate bleeding and the risk of erythrocyte necrosis within the clot, which can compromise the treatment outcome. To overcome these shortcomings, concentrated growth factor (CGF) has been introduced as an alternative scaffold (7). Another crucial factor influencing the success of RET is the elimination of canal infection. Persistent infection can damage intraradicular and periapical tissues and thereby decrease the success rate of treatment (8). Chemo-mechanical preparation is essential for the proper disinfection of the root canal system, particularly in areas inaccessible to instruments due to the complex root anatomy (9). Sodium hypochlorite, the gold-standard root canal irrigant, has antimicrobial properties as well as the ability to dissolve necrotic tissues and disrupt biofilms (10). However, using sodium hypochlorite in immature teeth is less successful than in mature teeth (11). Moreover, studies have shown that NaOCl can be cytotoxic and may adversely affect the stem cells of the apical papilla. Consequently, the American Association of Endodontists (AAE) advises irrigation with 1.5% sodium hypochlorite followed by 17% ethylenediaminetetraacetic acid (EDTA) in regenerative procedures (12 , 13). Given these challenges, alternative biocompatible methods are being actively explored. One promising approach involves the use of a biocompatible technique that delivers a substance rich in regenerative and antibacterial properties, derived from concentrated growth factor (CGF). Growth factors are bioactive proteins essential for regulating various cellular processes. CGF is an advanced second-generation platelet concentrate obtained through continuous differential centrifugation. It features a fibrin matrix embedded with growth factors, platelets, leukocytes, and CD34+ cells, all of which work synergistically to promote tissue regeneration. Furthermore, the immunological components of CGF help regulate inflammation and reduce the risk of infection (14). Alauddin et al. demonstrated that CGF possesses antimicrobial and anti-biofilm properties against pathogens such as Staphylococcus aureus and Streptococcus mutans (15). Although several studies have confirmed the antimicrobial properties of platelet derivatives, the underlying mechanism remains unclear. Possible explanations include the release of oxygen metabolites and ions, direct platelet-microorganism interactions, antimicrobial actions such as microbial aggregation and binding, and the presence of antioxidants and defense proteins, including defensin, lactoferrin, and cathelicidin (16). Melo-Ferraz et al. reported that platelet products such as leukocyte- and platelet-rich fibrin (L-PRF) inhibited pathogens, including Pseudomonas aeruginosa, Candida albicans, and Enterococcus faecalis. Both L-PRF membranes and exudates promoted platelet accumulation and biomarker production associated with wound healing and host defence (17). Controlling inflammation and proinflammatory cytokines, as well as eliminating endodontic pathogens, are crucial for the successful regeneration of the pulp-dentin complex. Therefore, this study aims to evaluate the antimicrobial efficiency of CGF against biofilms associated with endodontic diseases.

## Material and Methods

The study protocols were approved by the Research Ethics Committee of the School of Dentistry, Shiraz University of Medical Sciences (Approval ID: IR.SUMS.DENTAL.REC.1403.060, October 09, 2024). Written informed consent was obtained from all participants before sample collection. For each bacterial strain, the concentrated growth factor from each blood sample was split into two parts, resulting in three samples in the E. faecalis group and three samples in the C. albicans group. 1. CGF preparation Fresh venous blood samples (10 mL) were collected from 14 healthy, non-smoking volunteers with no systemic diseases, no signs of infection, and no history of antibiotic use in the preceding three months. All participants were fully informed about the study and provided written consent before sample collection. Blood samples were drawn in sterile anticoagulant-free glass tubes and immediately centrifuged using the following programmed cycle: 30 s acceleration, 2 min at 2700 rpm, 4 min at 2400 rpm, 4 min at 2700 rpm, 3 min at 3000 rpm, and 30 s deceleration to complete stop (PC-02, Process Ltd., Nice, France). Following centrifugation, four distinct layers were visible: the upper serum layer, a buffy coat, the CGF layer, and the red blood cells at the bottom. The CGF layer was aseptically removed using sterile tweezers and then separated from the surrounding layers with sterile microscissors. The clots were placed on a sterile gauze to remove the excess serum. Afterwards, the fibrin-rich CGF clots were minced, homogenized, and stored at -80°C for 1 hour. The samples were thawed and centrifuged at 3000 rpm for 10 minutes at room temperature. The resulting supernatant (CGF extract) was filtered through a sterile 0.22 μm syringe filter (Merck Millipore, CA) and stored at -80°C until use. 2. Minimum inhibitory concentration (MIC), minimum bactericidal concentration (MBC), and minimum fungicidal concentration (MFC) The MIC of CGF against E. faecalis (ATCC 29212) and C. albicans (ATCC 10231) was determined following the Clinical and Laboratory Standards Institute (CLSI) guidelines [18]. MIC is defined as the lowest concentration of an antimicrobial agent that effectively inhibits visible microbial growth. In brief, 100 μL of brain heart infusion (BHI) broth (Merck, Germany) was dispensed into each well of a 96-well microplate. Then, 100 μL of CGF was added to the first well of each row, serially diluted 1:2 across ten wells through repeated pipetting, and 100 μL was discarded from the final well. Subsequently, 100 μL of microbial suspensions (1.5 × 106 CFU/mL of E. faecalis and C. albicans) were separately added to each well. The microplates were then incubated at 37°C, and turbidity was visually assessed after 24 hours to determine the growth inhibition. MBC and MFC were determined by subculturing 2 μL from wells showing no visible growth onto BHI agar disks, followed by incubation at 37°C for 24 hours. The lowest concentration of CGF resulting in a 99.9% reduction in colony count was recorded as the MBC and MFC. 3. Disc agar diffusion assay (DAD) The DAD assay was performed according to the CLSI guidelines [18]. Microbial suspensions (~ 1.5 × 108 CFU/mL) were spread evenly on BHI agar plates. Sterile blank discs were impregnated with 20 μL of CGF at the MBC/MFC concentration and placed on the inoculated agar surface. The plates were incubated at 37°C for 24 hours. Afterwards, the diameters of the inhibition zones were measured. The culture medium served as the negative control, while 5.25% sodium hypochlorite (NaOCl) was used as the positive control. 4. Minimum biofilm inhibitory concentration (MBIC) The MBIC was determined as described by Macià et al. [19]. MBIC was defined as the lowest concentration of an antimicrobial agent resulting in an optical density (OD) at 650 nm differing by ≤10% (approximately a 1-log reduction after 6 h of incubation) from the mean of two positive control wells. At this concentration, there is no time-dependent increase in viable biofilm cell counts. Briefly, 100 μL of each microbial suspension (1.5 × 106 CFU/mL) was added separately to a 96-well microplate and incubated at 37°C for 24 hours to allow biofilm formation. Then, 100 μL of CGF (at the MBC/MFC concentration) was added with two exposure intervals (0 and 6 hours). Non-adherent cells and residual CGF were removed by washing with PBS. The biofilms were fixed with 100 μL of 95% ethanol for 10 minutes at room temperature (25 ± 2°C). The contents of each well were air-dried and stained with 200 μL of a 0.1% crystal violet solution for 15 minutes at room temperature. After three washes with PBS, 200 μL of 33% acetic acid was added to solubilize the dye. The OD was measured at 650 nm, and the percentage of biofilm inhibition was calculated as

(1) $$\text{Biofilm Inhibition (\%)}=(\frac{{\mathrm{OD}}_{\text{treat}}-{\mathrm{OD}}_{\text{control}(6h)}}{{\mathrm{OD}}_{\text{control}(6h)}-{\mathrm{OD}}_{\text{control}(0h)}}\times 100)-100$$

The negative control consisted of microbial suspension in culture medium, whereas the positive control consisted of wells treated with 5.25% NaOCl solution. 5. Minimum biofilm eradication concentration (MBEC) MBEC was determined according to Ceri et al. [20]. The MBEC is defined as the lowest concentration of an antimicrobial agent that destroys the biofilm. Microbial suspensions (200 μL, 1.5 × 106 CFU/mL) were added to 96-well microplates and incubated at 37°C for 24 hours to establish biofilms. 100 μL of CGF (MBC/MFC concentration) was added and incubated for 24 hours. After staining with crystal violet, OD at 650 nm was measured, and the percentage of biofilm eradication was calculated as.

(2) $$\text{Biofilm Eradication (\%)}=(\frac{{\mathrm{OD}}_{\text{control}}-{\mathrm{OD}}_{\text{treat}}}{{\mathrm{OD}}_{\text{control}}}\times 100)$$

6. Dental sample preparation Thirty intact premolars extracted for orthodontic purposes were collected. Longitudinal dentin sections were prepared from the middle third of each root in 2 mm thickness (4 × 4 mm) using a diamond disc. Samples were stored in physiological serum at room temperature until use. 7. Biofilm formation on dental samples To establish a microbial biofilm, 500 μL of each microbial suspension at a concentration of 1.5 × 106 CFU/mL was added to dental samples in the wells of a 24-well microplate containing BHI broth. The microplates were then incubated for 14 days at 37°C to perform biofilm formation on the dental samples, with the culture medium refreshed every 72 hours. 8. Viable microbial cell counting After treatment with CGF at MBIC and/or MBEC concentrations, the samples were rinsed with PBS to remove planktonic cells. Then, they were transferred to microtubes containing 1 mL of sterile BHI broth. Biofilms were detached by vortexing at high speed for 1 minute. Serial dilutions of these microbial suspensions were prepared in 96-well microplates, and 10 µL from each dilution was cultured onto BHI agar plates. The colony-forming units (CFUs)/mL for each treatment were determined following the method described by Miles et al. [21]. 9. Scanning electron microscopy (SEM) Two samples per group were randomly selected for SEM analysis to assess the disruption of biofilm structure or inhibition of growth on dentin surfaces after CGF treatment. 10. Statistical analysis The normality of data distribution was assessed before conducting one-way ANOVA. Post-hoc pairwise comparisons were performed using Tukey’s test. Statistical significance was defined as p<0.05, with a 95% confidence interval. The data are presented as mean ± standard deviation (SD) and analyzed using GraphPad Prism software (version 8.4.3). All assays (MIC, MBC/MFC, DAD, MBIC, and MBEC) were performed in triplicate and repeated three times independently, while CFU counts were repeated five times for accuracy and reproducibility.

## Results

**Figure 1:**
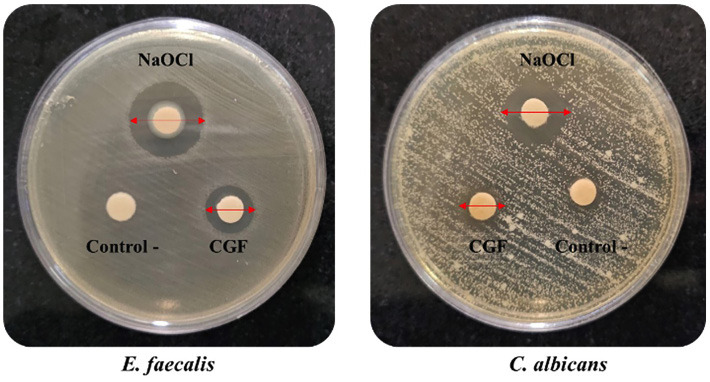
Growth inhibition zones produced by CGF and NaOCl against E. faecalis and C. albicans after 24 h of incubation. CGF (100%v/v) produced smaller zones than NaOCl (Scale=10 mm).

1. MIC, MBC, and MFC The MIC of CGF against both microorganisms was 50% v/v, while the MBC and MFC reached 100% v/v. These values indicated that a 50% v/v concentration of CGF effectively inhibited microbial growth, however, a full 100% v/v concentration was required to eliminate the microorganisms. The difference between the MIC and MBC/MFC suggests that CGF can inhibit microbial growth at lower concentrations, while a complete eradication of the microbial cells requires a higher concentration. This demonstrates its potential effectiveness as an antimicrobial agent, depending on whether the goal is to inhibit or eliminate microbial growth. 2. Disc agar diffusion assay As shown in Figure 1, a growth inhibition zone was observed around the CGF on the E. faecalis and C. albicans plates.

1

Table 1 presents the average diameter of the growth inhibition zone (in mm) for CGF and NaOCl.

Table 1

3. Anti-biofilm effects of CGF against E. faecalis and C. albicans According to the findings, the MBIC of CGF for both E. faecalis and C. albicans was 100% v/v. Regarding the MBEC of CGF, a complete eradication (99.9%) was not observed for E. faecalis or C. albicans. These results suggested that while CGF inhibited biofilm formation at a concentration of 100% v/v, a complete biofilm eradication (99.9%) was not achieved for E. faecalis or C. albicans under the tested conditions. The results also showed that 5.25% NaOCl significantly inhibited microbial biofilm formation and effectively eradicated pre-formed biofilm (Tables 2,3).

Table 2

Table 3

**Figure 2:**
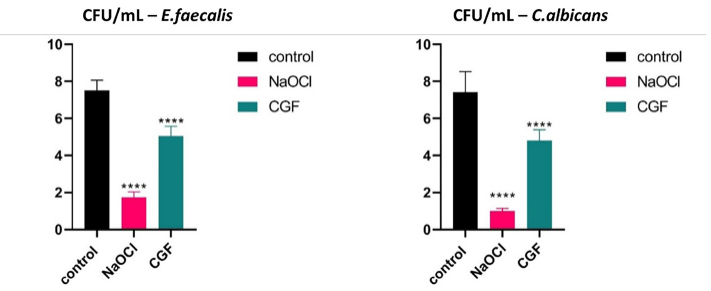
Viable cell counts (CFU/mL) of E. faecalis and C. albicans after treatment with CGF and NaOCl. Statistical analysis was performed using one-way ANOVA with Tukey’s post-hoc test (****p &lt; 0.0001 compared with the control).

4. Determination of viable cell numbers of biofilms cultured on tooth slices In this study, 100% v/v of CGF was used to further analyze its antibiofilm activity on tooth slices. The mean E. faecalis viable cell counts (×105 CFU/mL; n=5) were 7.52 ± 0.54 in the control, 5.04 ± 0.53 after CGF treatment, and 1.74 ± 0.30 after NaOCl treatment. Mean C. albicans CFU counts (×105 CFU/mL; n=5) were 7.42 ± 1.11 (control), 4.80 ± 0.59 (CGF), and 1.00 ± 0.15 (NaOCl). The results showed a significant reduction in viable microbial cells after the treatment of E. faecalis and C. albicans biofilms with NaOCl and CGF compared to the control group (p&lt;0.0001; Fig. 2).

2

**Figure 3:**
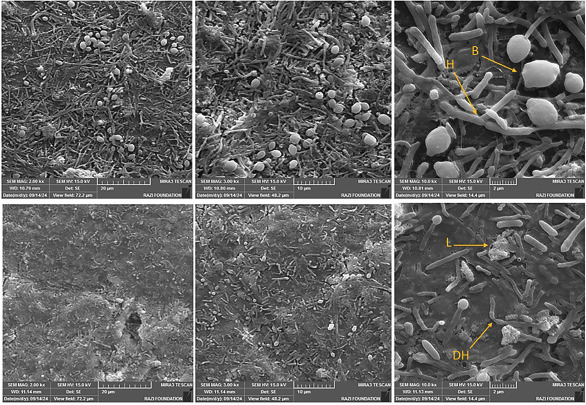
The SEM micrographs showing Candida Albicans biofilms on the dentin surface with (top row) and without treatment with CGF (bottom row). The images were taken at 2.00kx, 3.00kx, and 10.0kx magnification from left to right. Untreated controls show a dense structured biofilm with abundant hyphal elements (H), blastospores (B), and extracellular matrix covering the dentin surface. CGF treatment reduced biofilm coverage relatively, disrupted biofilm architecture, and resulted in disrupted hyphae (DH) and lysed cells (L) with visible debris, leaving areas of exposed dentin.

There were 76.86% and 86.52% reductions in viable microbial cells following the NaOCl treatment of E. faecalis and C. albicans biofilms, respectively. Additionally, CGF treatment resulted in a 32.97% reduction in E. faecalis biofilm and a 35.30% reduction in C. albicans biofilm. 5. Evaluation of biofilm topography by Scanning Electron Microscopy (SEM) The SEM results in Figure 3 show clusters of ovoid and spherical fungal cells of C. albicans embedded in dense extracellular material in a control growth medium, along with evidence of bud scars at the cell poles.

3

**Figure 4:**
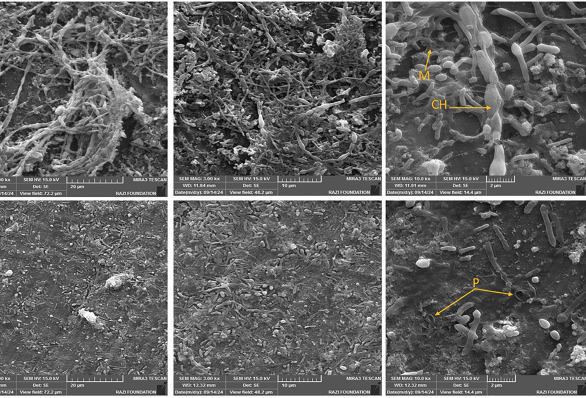
The SEM micrographs showing E. faecalis biofilms on the dentin surface with (top row) and without treatment with CGF (bottom row). The images were captured at 2.00kx, 3.00kx, and 10.0kx magnification from left to right. Untreated controls display dense multilayered biofilms with extensive surface coverage, featuring intact chains of cells (CH) embedded in an extracellular matrix (M) that uniformly covers the dentin surface. Following CGF treatment, the micrographs show reduced biofilm coverage, leaving large areas of exposed dentin. High-magnification images reveal cells with surface pores (P) and irregular morphology, indicating damage to the cell wall and disruption of the biofilm.

After 24 hours of treatment with CGF, there was a significant reduction in fungal cell density. The remaining damaged cell structures indicated that the microorganism had faced challenging conditions. The SEM results of the control group, shown in Figure 4, revealed a structured E. faecalis biofilm with irregular microgranular bacterial clumps on the dentin surface.

4

However, after treatment with CGF, the growth of the adhered cell clumps was disrupted, and a much smaller bacterial population was observed.

## Discussion

Endodontic infections are primarily caused by microorganisms invading the root canal system. Among fungi, Candida albicans is the most frequently isolated species. It adheres to dentin, penetrates dentinal tubules, and forms biofilms that resist intracanal disinfectants, allowing it to persist despite endodontic treatment (22). Its morphological plasticity, specifically the ability to switch between yeast and hyphal forms, aids immune evasion and robust biofilm development (23). E. faecalis is a facultative anaerobic gram-positive coccus that adapts well to the root canal environment, which is nutrient-poor, has low oxygen levels, and has a complex ecology (24). Laboratory studies have demonstrated that E. faecalis exhibits a notable resistance to endodontic treatment procedures, partly due to its ability to form biofilms and survive under harsh conditions. This resistance is attributed to a cell membrane-bound proton-transport system, which is essential for survival at extreme pH levels, as in Ca(OH)2 treatment (25). In the current context, regenerative endodontic treatment (RET) is widely preferred for immature permanent teeth diagnosed with pulp necrosis or apical periodontitis (26). RET allows continued root development, resulting in the thickening of the dentinal wall. This, in turn, leads to an improved fracture resistance and a better long-term prognosis (27 , 28). The success of RET is critically dependent on an effective root canal disinfection, as residual infection impairs the regeneration processes and negatively influences stem cell survival and differentiation (29). Sodium hypochlorite (NaOCl) remains the gold standard irrigant in RET because of its potent antimicrobial activities and ability to dissolve necrotic tissue, achieved through mechanisms such as membrane disruption, protein denaturation, and oxidizing activity (30 , 31). However, NaOCl is cytotoxic to stem cells at higher concentrations and may compromise regenerative potential (32). Our findings suggest that CGF can significantly inhibit biofilm formation by E. faecalis and C. albicans although it is unable to eradicate mature biofilms completely. This antimicrobial effect is consistent with previous reports on platelet concentrates, such as platelet-rich plasma (PRP) and platelet-rich fibrin (PRF), which have exhibited antimicrobial activity against oral pathogens including E. faecalis and C. albicans. This effect has been largely attributed to their platelet content and release of antimicrobial peptides such as defensins and -defensins (16 , 33 , 34). The bioactivity of CGF is likely attributable to its enriched composition of leukocytes, platelets, and peptides (defensins, lactoferrin, platelet microbicidal proteins), as well as enzymes such as myeloperoxidase and phospholipase A2 (35). Additionally, CGF retains plasma-derived complement proteins that may contribute to microbial lysis and leukocyte recruitment (36). Through these combined mechanisms, CGF not only exerts a moderate antimicrobial activity but also provides a fibrin scaffold capable of sustained release of growth factors, such as VEGF, TGF-1, and PDGF, which support angiogenesis, wound healing, and odontogenic differentiation (37 - 39). Unlike NaOCl, which primarily functions as a chemical disinfectant, CGF combines antimicrobial activities with biological properties that favor stem cell survival and tissue regeneration (40). Centrifugation during CGF preparation enriches the final product with leukocytes, platelets, antimicrobial peptides, and enzymes that enhance its antimicrobial potential. Platelets can detect, target, and eliminate microorganisms by releasing reactive oxygen metabolites and antimicrobial peptides (41 , 42). In the present study, CGF inhibited biofilm formation by E. faecalis and C. albicans. However, these findings should be interpreted cautiously. They are based on an in vitro model using single-species biofilms and planktonic suspensions. Such a model cannot fully replicate the structural heterogeneity of polymicrobial communities or their interactions with host immune defenses in vivo. As a scaffold enriched with growth factors, CGF may persist longer in the root canal system. It may also support the viability of odontogenic stem cells, thereby creating a biologically favorable environment for tissue repair. These are effects that conventional irrigants alone cannot achieve (43). These properties, combined with the ability to modulate inflammation and promote odontogenic differentiation, suggest that CGF may serve as a valuable adjunct in RET. Still, further in vivo and clinical investigations are required to confirm its antimicrobial efficacy and establish optimized protocols for clinical application.

## Conclusions

According to the results of the present study, CGF demonstrated moderate antimicrobial and antibiofilm activity in vitro against E. faecalis and C. albicans. By inhibiting biofilm formation, CGF may serve as a bioactive adjunct in regenerative endodontic therapy, supporting tissue repair while preserving the viability of stem cells. However, its antimicrobial effects are moderate and insufficient in eliminating mature biofilms completely. Further in-depth in vivo and clinical studies are required to confirm these findings and establish optimal protocols for incorporating CGF into regenerative endodontic procedures.

## Figures and Tables

**Table 1:** Mean diameter of the growth inhibition zones (mm) of E. faecalis and C. albicans treated with CGF and NaOCl.

Groups	Diameter of the growth inhibition zone (mm)	
	E. faecalis	C. albicans
Control	No inhibition	No inhibition
NaOCl	20	18
CGF (100% v/v)	9	8

1

**Table 2:** Mean (±SD) optical density values showing MBIC against E. faecalis and C. albicans (two-way ANOVA with Tukey’s post-hoc test).

E. faecalis	Control 0h	Control 6h	CGF	NaOCl
Mean ± SD	0.38 ± 0.07	0.52 ± 0.06	0.37 ± 0.06	0.18 ± 0.03
C. albicans	Control 0h	Control 6h	CGF	NaOCl
Mean ± SD	0.59 ± 0.01	0.71 ± 0.08	0.65 ± 0.06	0.44 ± 0.04

2

**Table 3:** Mean (±SD) optical density values showing MBEC against E. faecalis and C. albicans (two-way ANOVA with Tukey’s post-hoc test).

E. faecalis	Control	CGF	NaOCl
Mean ± SD	1.36 ± 0.20	0.70 ± 0.05	0.08 ± 0.02
C. albicans	Control	CGF	NaOCl
Mean ± SD	1.63 ± 0.05	0.61 ± 0.09	0.11 ± 0.02

3

## Data Availability

The authors will make the raw data supporting the conclusions of this article available upon request.
